# Soft-stable interface in grasping multiple objects by wiring-tension

**DOI:** 10.1038/s41598-023-47545-3

**Published:** 2023-12-06

**Authors:** Pho Van Nguyen, Dhyan Bohra Sunil, Wai Tuck Chow

**Affiliations:** 1 School of Mechanical and Aerospace Engineering, Nanyang Technological University, Singapore, Singapore; 2 Schaeffler Hub for Advanced Research Center (SHARE@NTU), Singapore, Singapore

**Keywords:** Electrical and electronic engineering, Mechanical engineering

## Abstract

Efficiently manipulating objects in a group state poses an emerging challenge for soft robot hands. Overcoming this problem necessitates the development of hands with highly stable structures to bear heavy loads and highly compliant designs to universally adapt to various object geometries. This study introduces a novel platform for the development of robot hands aimed at manipulating multiple objects in each trial. In this setup, the objects come into soft contact with an elastic wire affixed to the finger skeletons. This combination results in a harmonious hybrid finger, inheriting both the soft, flexible properties of the wire and the robust stability provided by the finger skeleton. To facilitate this approach, a theoretical model was proposed to estimate the kinematics of manipulating multiple objects using wiring-based fingers. Based on this model, we designed a hybrid gripper comprising two wiring-based fingers for conducting experimental evaluations in manipulating four groups of samples: a pair of bevel gears, a pair of bevel gears plus a pneumatic connector, a pair of glue bottles, and a pair of silicon bottles. The experimental results demonstrated that our proposed gripper reached good performance with high success rates in durability tests conducted at various lifting velocities and high adaption with objects in soft-friendly ways. These findings hold promise for efficiently manipulating multiple complex objects in each trial without the need for complex control systems.

## Introduction

Previous works on robotics grippers focused mainly on mimicking living organisms or replicating other traditional mechanisms which can be found in many actual applications^1–5^ and in agriculture^6^. For instance, a gecko-like pad^7^ attached to the soft finger or a magnetic-driven composite pad^8^ were useful for manipulating objects in dry environments with low squeeze requirements. Handling dry objects was also demonstrated by a jamming gripper with a dielectric elastomer embedded on the contact interface^9^. The work done by Hawkes and Jun^7–9^ offered advantages to manipulate dry objects, however, they had limitations to bear heavy loads due to their very soft bodies. Furthermore, the application of high voltage for actuation could pose operational risks and potential damage. In contrast, stronger soft robot hands mimicking an octopus arm^10–12^, fin ray gripper^13,14^, compliant grippers^15–17^, and jamming grippers^18,19^ can flexibly hold large objects by utilizing suction and frictional forces generated by their bodies. Other soft robotic hands in^20–25^ showed their benefits in grasping wet materials such as the food samples thanks to the increment of the wet adhesion force generated by a tree-frog inspired pad or wrinkle structure^26,27^. Despite the progress in soft robotic hands for manipulation, two main challenges persist: the inherent low stability of their body structures and the issue of infinite degrees of freedom (DoFs). These challenges limit the soft grippers’ ability to achieve high-performance levels when manipulating multiple objects in each trial.

In terms of high stability in manipulation, conventional grippers constructed by two or three rigid-linkage fingers in^28–30^ present themselves as strong candidates when compared to soft grippers. Prosthetic hands with five rigid-phalanx fingers actuated by a cable-driven system^31–33^ also presented advantages in heavy objects with high flexibility. Traditional robot hands, characterized by high-stiffness fingers, rigid phalanxes, and finite degrees of freedom (DoFs) at each joint, excel in controlling grasping poses and securely locking group objects with a consistent squeeze. These attributes are advantageous for rapidly manipulating objects with high production efficiency. Furthermore, this type of gripper can adapt to a wider range of objects, which overcomes the drawback of soft grippers, which are inherently suitable for specific one or a few objects. However, due to generating unfriendly interactions, traditional grippers may yield unexpected outcomes, especially when dealing with soft-fragile objects. Additionally, the finite DoFs become a barrier, significantly restricting the flexibility of such grippers in approaching and manipulating group objects. The rigid fingers in such grippers were improved by integrating granular structure on the finger phalanx^34^ or and the flexible knuckles actuated by the heating elements^35^ to generate the soft interaction and more flexibility. Our previous works introduced a hybrid gripper comprising two array fingers working as a claw comb^36,37^. Each finger was printed with hard materials and covered with a thin silicon film on the surfaces that directly contacted the objects. While our gripper could grip many objects at each trial compared with soft Pneunet grippers, some objects still dropped due to the large vacant space inside the two claws. In addition to achieving universal manipulation, the ability of a gripper to handle multiple objects in a single manipulation trial is a practical concern that significantly contributes to improving labor productivity. Authors in^38^ came up with an analytical model for the rigid gripper in picking up multiple objects per trial. Other groups in^39–41^ and^42–46^ recently have started to seek learning models relating to manipulating multiple objects by one gripper. Those presented works aimed to develop the robot hand in handling multiple-object, however, they concentrated on setting up the algorithms and theory models, whereas the safety and protecting the objects during manipulations have not been mentioned.

This paper introduces a novel platform to target the universal manipulation of multiple objects with soft interaction while maintaining stability. In this platform, multiple objects are initially placed on the floor in either sparse or cluster arrangements before the robot fingers approach, collapse, grasp and lift them away. At each trial, the robot fingers try to successfully manipulate at least two objects (identical or mixing objects) with help of the squeezing from an elastic wire mounted to a finger skeleton, aka wiring-based finger. The wire facilitates the group objects to self-arrange until they reach a balanced state. Concurrently, the finger skeleton controls the wiring tension to fasten the objects. The hybrid finger, which combines the wire and the finger skeleton, is incorporated into a mathematical model for estimating the kinematics of handling the object, and the design of a novel robot gripper (denoted as a wiring-based gripper) having two symmetrical fingers. Experimental trials were then conducted to assess the performance of this gripper in manipulating multiple objects during each trial. The input parameters for these experiments were also verified using mathematical equations to validate the predictions. The novelties proposed in this paper include a novel platform for developing robot grippers that utilize wire-based fingers to manipulate multiple objects in a single trial and a theoretical model to investigate the mechanics of wire-based fingers when grasping objects.

**Figure 1:**
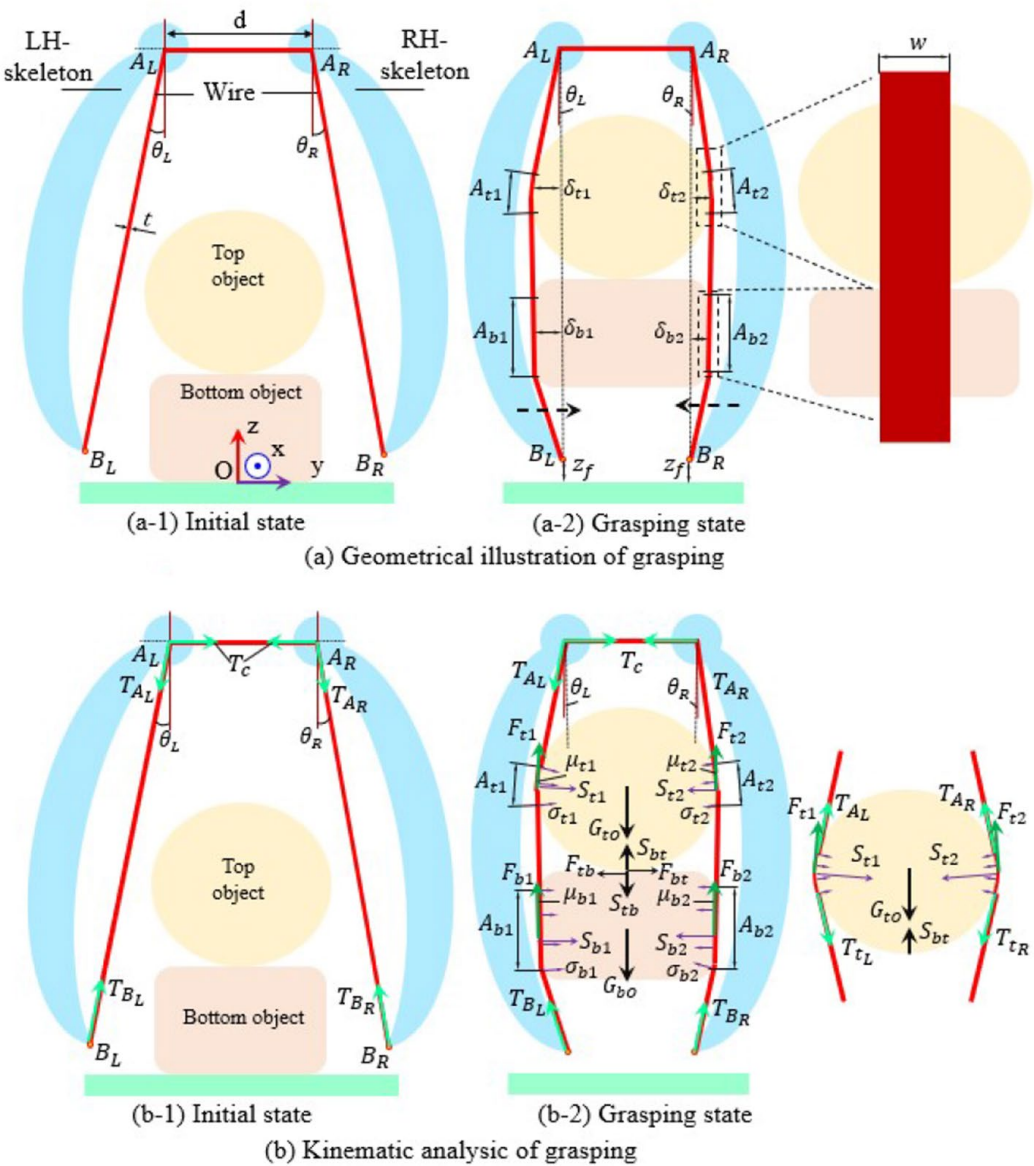
Principal illustration of grasping two objects by two wiring-based fingers with (**a**) geometrical analysis, and (**b**) kinematic analysis before grasping (**a-1**), and (**b-1**) and after grasping (**a-2**), and (**b-2**). Black dash arrows indicate the moving directions of each finger.

**Figure 2:**
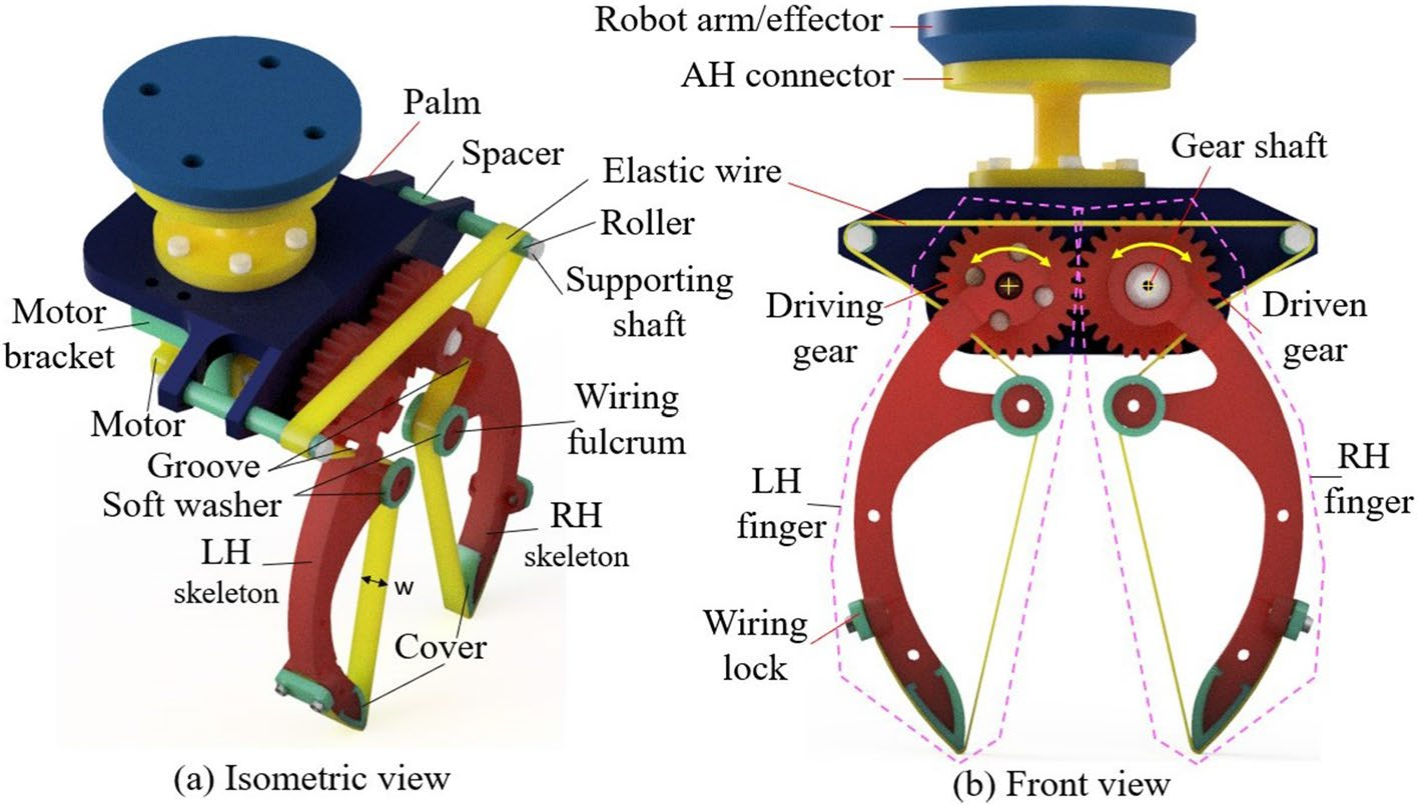
Three-dimension design of the wiring-based gripper in isometric view (**a**) and front view (**b**). Each finger was packaged inside a pink dash loop, concurrently, yellow arrows and crosses illustrated rotating directions and rotating center of each finger.

## Design and method

### Theoretical model of grasping with elastic wire

In Fig. 1, the mathematical model comprises a pair of wiring-based fingers symmetrically positioned outside of the objects placed on the floor. Each finger comprises one elastic wire pre-stretched on a finger skeleton. One wire has the width, and thickness *w*, *t*, and its two ends are fixed at $B_L$ and $B_R$. Two finger-skeletons are rigid bodies that can rotate around the fixed center $A_L$ and $A_R$ with the distance *d*. When two fingertips move towards each other, the wiring length $l_w$ changes at $A_L$ and $A_R$. Concurrently, as the wire contacts the objects: top and bottom at four contact positions $A_{t1},A_{t2},A_{b1},A_{b2}$, the wire has the lateral deflections $\delta _{t1},\delta _{t2},\delta _{b1},\delta _{b2}$ at such positions, respectively. Assume that the minimum length of the wire *min*$\{ l_w \}$ is the sum of $d+2A_LB_L$, the variation of $l_w$ depends on its variations at $A_L$, $A_R$, and the contact positions. In other words, the variation of wiring length ($\delta l_w$) is estimated as follows:

1 $$\begin{aligned} \varvec{\delta l_w=\delta l_w(A_L,A_R)+\delta l_w(\delta _{t1})+\delta l_w(\delta _{t2})+\delta l_w(\delta _{b1})+\delta l_w(\delta _{b2})}. \end{aligned}$$

In Eq. (1), $\delta l_w$ is a function of $\varphi _L,\varphi _R$ and the properties of the objects such as shape, size, weight, and so on. Such parameters require the wire to have enough wiring tension $T_w$ for holding the objects by generating lateral deformations. In Fig. 1a, $\delta l_w$ is direct covariate with $\delta _{t1},\delta _{t2},\delta _{b1},\delta _{b2}$ which can be determined by the kinematic analysis.

At the minimum value of $l_w$, we have the initial condition relating to the wiring tension as follows: $T_{B_L}=T_{A_L}$, $T_{A_R}=T_{B_R}$, $T_{A_L}=\xi _L T_c$, and $T_{A_R}=\xi _R T_c$. In this scenario, $\xi _{R,L}$ are the functions relating to the structure design at $A_L$ and $A_R$. In terms of the same $\xi _{R,L}$, the wiring tension at $B_L$ equals to at $B_R$. Concurrently, if $\xi _{R,L}\rightarrow 1$, the wiring tension $T_w$ is unchanged at each point on the wire. At the grasping state, the fingers gradually close until the objects are located inside the wire. The objects are gripped and held due to the squeeze from the wiring tension. At each contact position, $T_w$ generates the squeeze forces $S_{t1},S_{t2},S_{b1},S_{b2}$ which are the integral of the normal stress on the contact surfaces $\sigma _{t1},\sigma _{t2},\sigma _{b1},\sigma _{b2}$. This kind of force creates the friction force $F_{t1},F_{t2},F_{b1},F_{b2}$ between the objects and the wire. When the gripper lifts up, the friction has to be larger than the total gravity of the objects to prevent objects from falling down. Additionally, the squeeze and friction force exists at the contact position between the top and bottom objects (aka $S_{bt},S_{tb},F_{bt},F_{tb}$). The total force and moment acting on each object in plane *Oyz* is zero to achieve the stable picking up. In other words, the total reaction forces on each object satisfy the following equation:

2 $$\begin{aligned} {\left\{ \begin{array}{ll} \varvec{(\sum F + \sum S)_y = 0} \\ \varvec{(\sum F + \sum S)_z = kG} \\ \varvec{\sum F d_F + \sum S d_S = 0} \end{array}\right. } \end{aligned}$$

In Eq. (2), ${\textbf {F}}$ and ${\textbf {S}}$ are respectively the matrix of friction and squeeze forces, $d_F$ and $d_S$ are the distances from the object’s center to the force vectors ${\textbf {F,S}}$. Also, $()_y$ and $()_z$ are the projecting of the entities on *y* and *z* axes. This study focuses on picking up the objects in *z* direction, the safety factor *k* is larger than 1. At the balance state, we majorly focus on the equivalent condition in Eq. (2), whereas the remaining conditions can be ignored due to the symmetrical design. Let us denote $\mu$ = [$\mu _{t1},\mu _{t2},\mu _{b1},\mu _{b2}$] is the frictional coefficient matrix, the friction force **F** is a proportional entity with the squeeze force **S** by $\mu$. Hence, the middle condition in Eq. (2) can be rewritten in Eq. (3):

3 $$\begin{aligned} \varvec{(\sum S)_z = \frac{kG}{1+\mu } }. \end{aligned}$$

Determining **S** in Eq. (3) is based on the wiring tension at the contact positions. For instance, Fig. 1b shows the kinematics model at the contact on the top object where we have: $(S_{t1})_y$ = $-(T_{A_L}+T_{t_L}+\mu _{t1}S_{t1})_y$, $(S_{t1})_z$ = $(T_{A_L}-T_{t_L}+\mu _{t1}S_{t1})_z$, $(S_{t2})_y$ = $-(T_{A_L}+T_{t_L}+\mu _{t2}S_{t2})_y$ and $(S_{t2})_z$ = $(T_{A_L}-T_{t_L}+\mu _{t2}S_{t2})_z$. Hence, we can get the relation between the squeeze and the wiring tension in Eq. (4):

4 $$\begin{aligned} \varvec{S \approx \frac{1}{(1-\mu )}\sqrt{(2T_c+\delta T_c)^2_y + (\delta T_c)^2_z}} \end{aligned}$$

with $\delta T_c$ as the deviation of the wiring tension when the wire contacts the external surfaces such as the objects. $T_c$ depends on the length of wire $l_w$. In case of small $\delta l_w$, $T_c$ can be calculated as shown in Eq. (5):

5 $$\begin{aligned} \varvec{T_c = T_{c,min}+\frac{\delta l_w Y_w w t}{l_{w,min}}} \end{aligned}$$

with $Y_w$-Young modulus of the wire and $T_{c,min}$-the wiring tension at the minimum length of the wire $l_{w,min}$.

### Design wiring-based gripper

**Figure 3:**
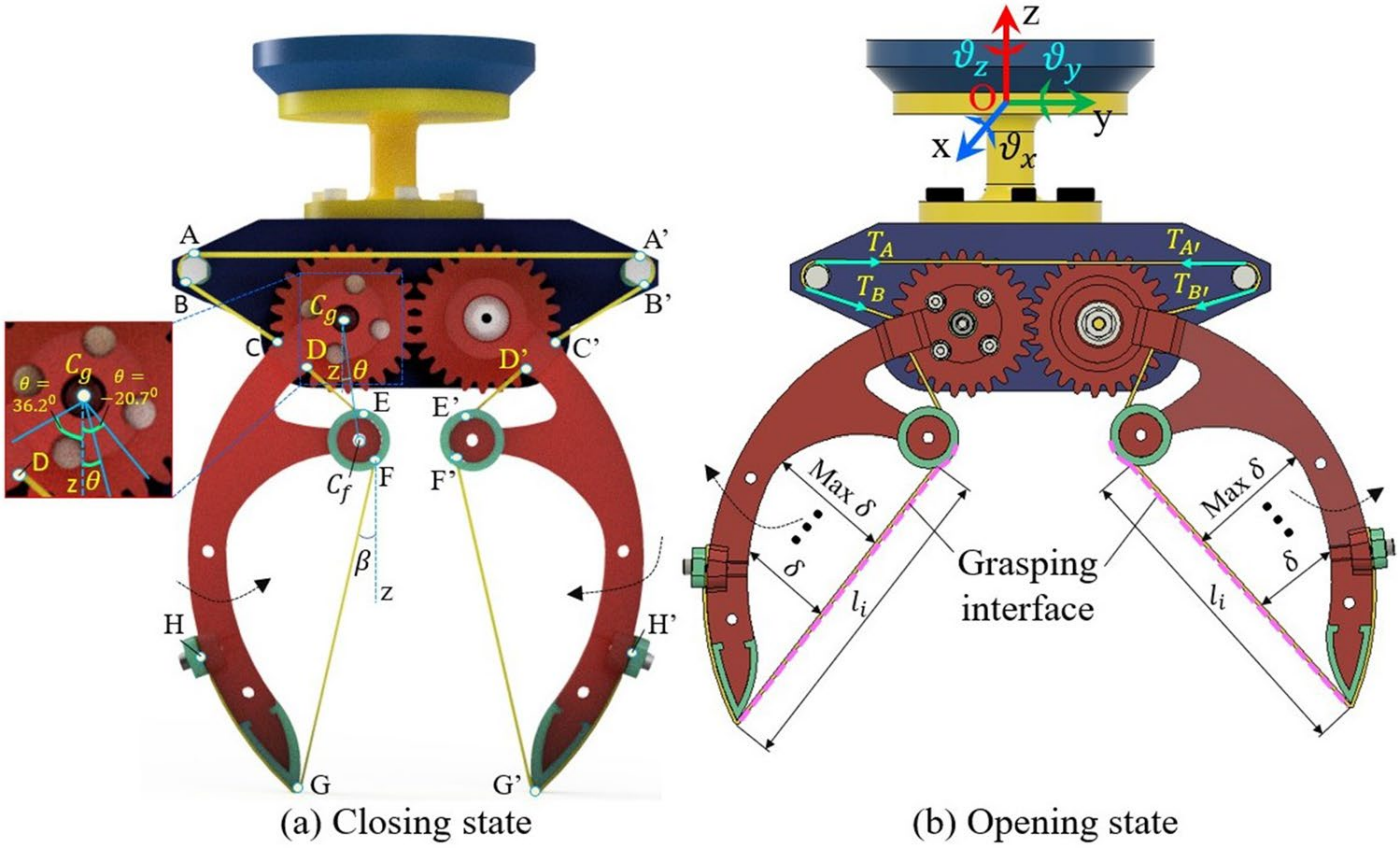
Principle illustration of wiring-based gripper at closing state (**a**) and opening state (**b**). A, B, A′, and B′ are the contact points between the wire with the supporting shaft, C, D, E, F, G, C′, D′, E′, F′, and G′ are the contact points between the wire with the skeleton and H, H′ are the terminated points of the wire at the wiring lock.

In this section, the wiring-based fingers were designed based on Fig. 1. In Fig. 2, the shape of skeletons is a curve to increase the storing volume and facilitates the large deflection of the wire. The fingertip is sharp, which makes it easy to creep into the narrow gaps when grasping objects among multiple items. A fulcrum in the skeleton changes the angle $\beta =\widehat{FG,z}$ (see Fig. 3a). The end of each skeleton has a gear in which the driving gear and driven gear belong to the LH- and RH skeletons, respectively. The driving gear directly binds to a rotating shaft of a servo motor located under the palm, while the driven gear rotates around its center shaft which is fixed to the palm. At the front view, if the rotating shaft turns anti-clockwise or clockwise, the fingers simultaneously close or open, respectively. The wire originated on a wiring lock of the LH-skeleton and traversed through the fingertip, wiring fulcrum, and supporting shaft (on the left side of the palm). Continuously, it is symmetrically mounted for the right-hand side of the palm and the RH skeleton before ending at another wiring lock. The upper surface of the palm fixes to an arm-hand connector (aka AH connector), a linking component for attaching the robot hand with other end effectors such as the robot arm. In Fig. 3, the lengths of the wire at each line/curve are $l_1 =AA'$, $l_2=AB$, $l_3=CD$, $l_4=DE$, $l_5=FG$, $l_6=GH$, and $\theta =\widehat{C_gC_f,z}$. Also, the length of the entire wire is computed by:

6 $$\begin{aligned} l_t = l_1 + 2\sum _{n=2}^{6} l_n. \end{aligned}$$

The variation of $l_t$ in Eq. (6) causes the change of the wiring tension in Eq. (5). The palm and the finger’s skeletons of the wiring-based robot hand in Figs. 2, and 3 were printed in a Prusa 3D printer. The soft fingertip and the soft fulcrum were covered by the silicon washers and covered with a thin-glue film. A rubber band representing the wire was mounted on the finger skeletons following the trajectory in Fig. 3. DSSERVO Servo Motor 35KG Coreless was mounted to drive the LH finger.

### Experimental setup

**Figure 4:**
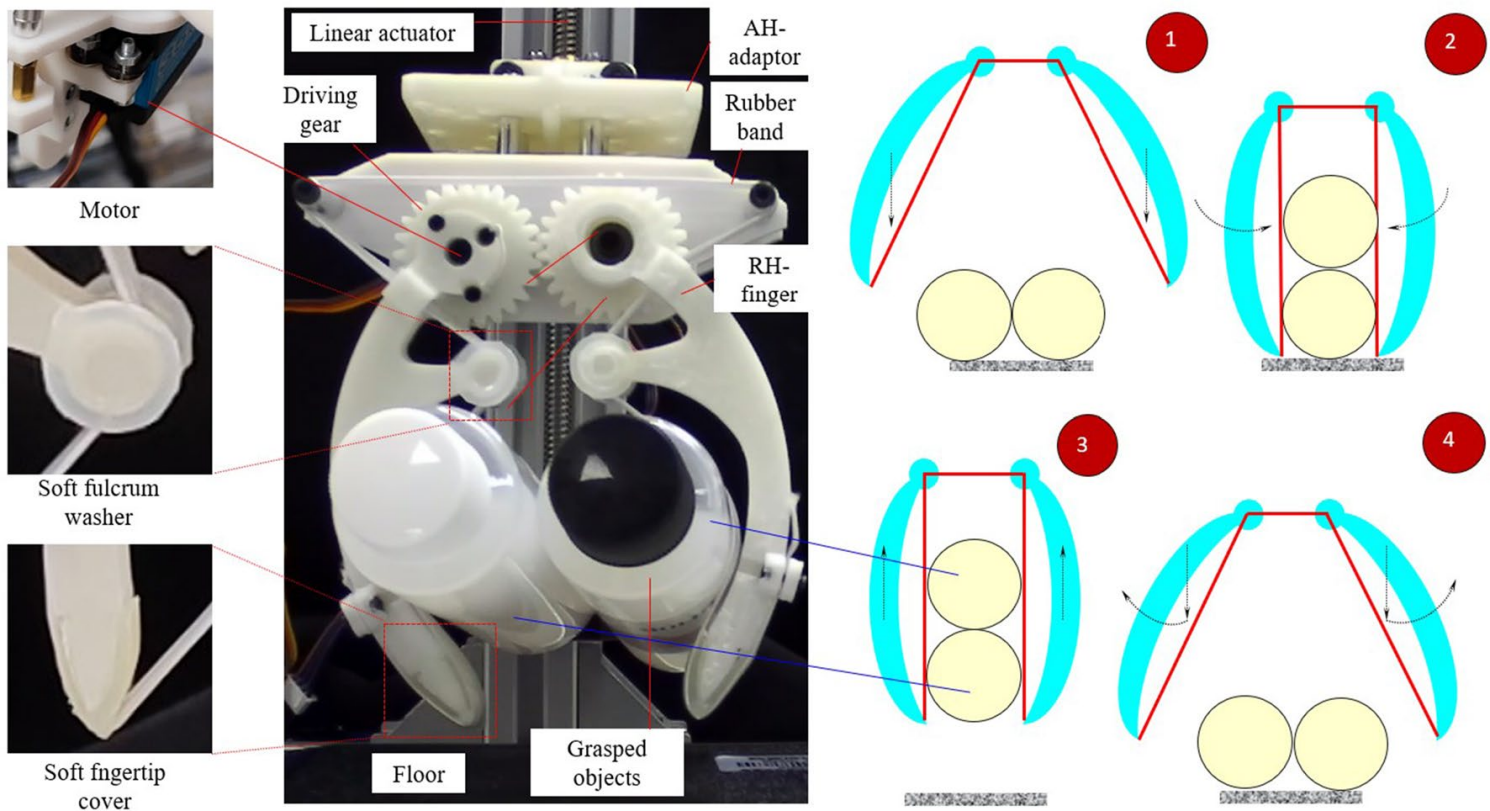
Schematic illustration of the experimental setup with four steps: 1—approaching, 2—grasping, 3—lifting, and 4—releasing. The dash-line arrows show the translating and rotational motion of the fingers at each step.

The fabricated gripper in Fig. 3 was fixed to a linear actuator having *z*-axis motion for evaluating the grasping performance with multiple objects (see Fig. 4). In this experiment, the screw of a linear actuator was directly actuated by a Bachin stepper 4240-15A controlled by an Arduino Uno board. Initially, the objects were placed on the floor and the fingertips opened and were set up with higher positions than the highest apexes of such grasped objects. We categorized the objects into two groups: *C1*—hard objects: double bevel gear (mass 24 g each), and double bevel gear (mass 24 g each) plus a pneumatic connector (mass 5.8 g each) and *C2*—soft objects: double glue bottles (mass 10 g each), and double silicon bottles (mass 120 g each). At each experiment in Fig. 4, the objects in groups *C1*, and *C2* were always manipulated in the group (at least two items were picked up per trial). Each experiment trial is sequentially spent through 4 phases. (1) *approaching*: a pair of fingers maximum opened and then moved down. (2) *grasping*: these fingers are anti-clockwise rotated to grasp the objects. (3) *lifting*: the gripper continuously grasped and lifted the grasped object up with 40 mm. Finally, (4) *releasing*: after stopped 5 s, the hand moved down to its initial position and dropped grasped objects to the floor. Additionally, each trial was carried out at three slide velocities $v_z$ of 0.57, 1.08, and 3.63 cm/s, sequentially. At each trial, 10 iterations were conducted to validate the gripper’s durability in grasping.

### Performance of gripper in handling various objects

**Figure 5:**
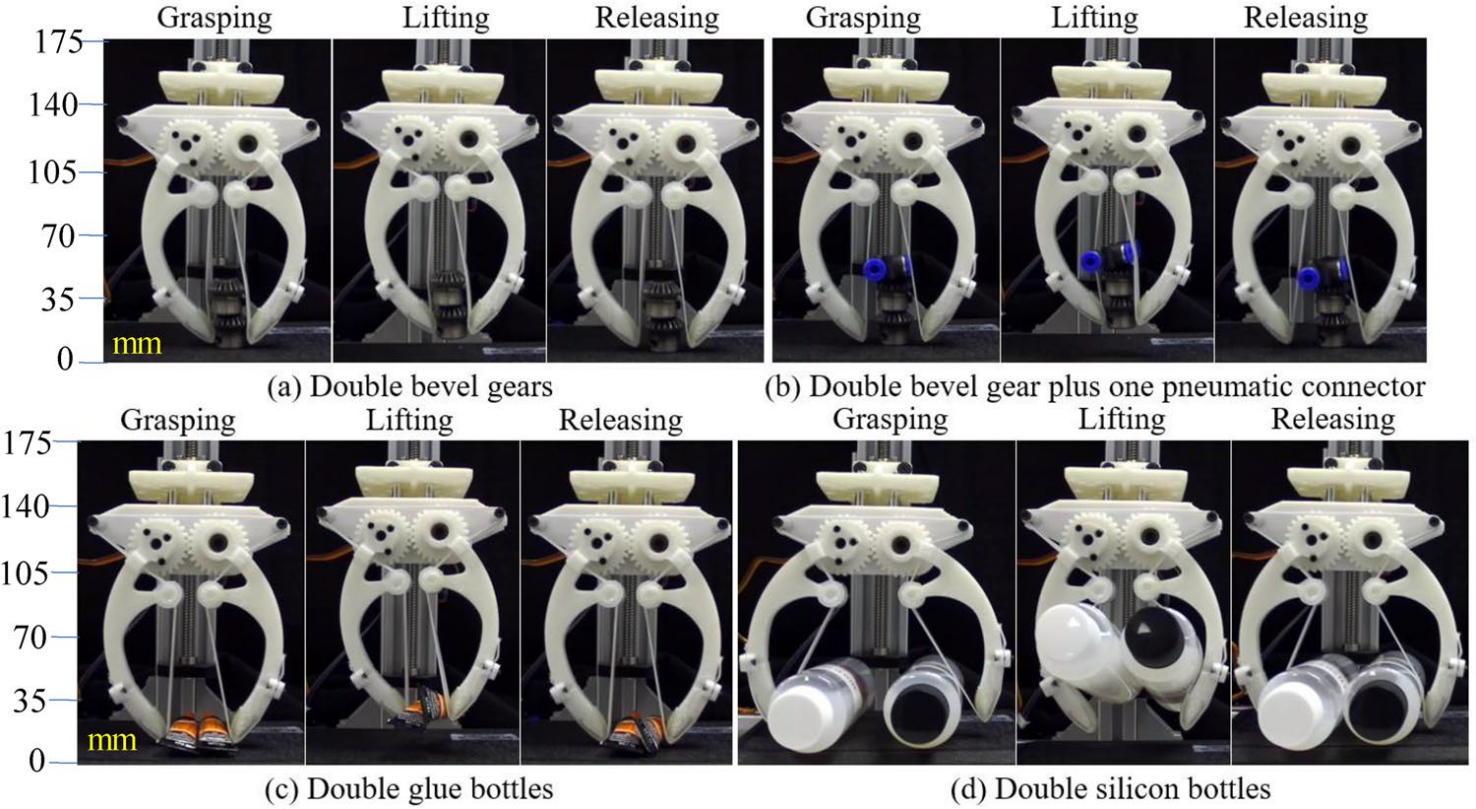
Demonstration of the wiring-based robot hand in gripping and lifting multiple objects in group *C1*, and *C2*: (**a**) double bevel gears, (**b**) double bevel gears plus a pneumatic connector, (**c**) double glue bottles and (**d**) double silicon bottles.

**Figure 6:**
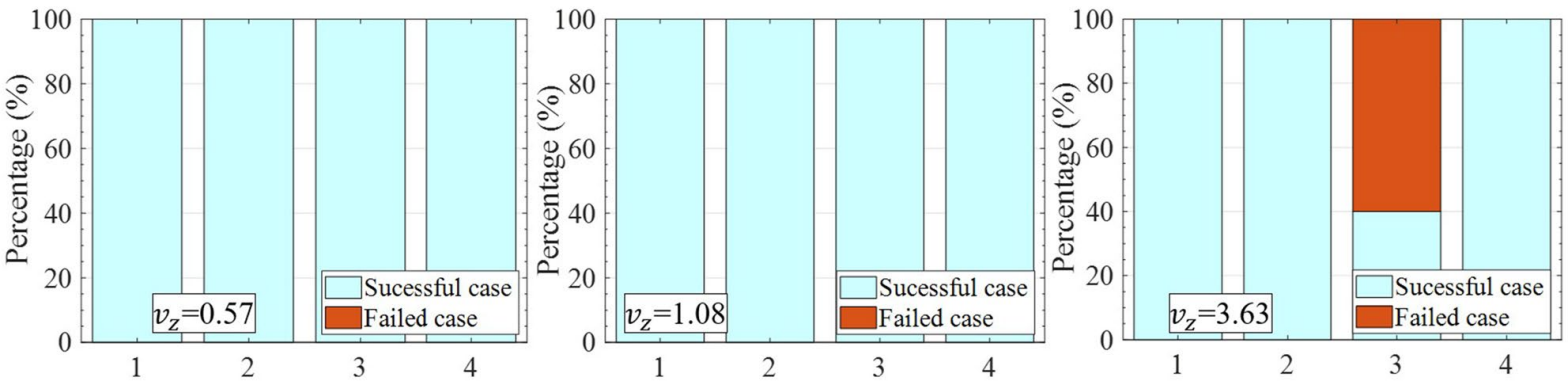
Durability test in gripping multiple objects per trial by the wiring-based robot hand under 3 levels of $v_z$ = 0.57 cm/s, 1.08 cm/s, and 3.63 cm/s. 1, 2, 3, 4 were respectively double bevel gear, double bevel gear+pneunet connector, double glue bottle, and double silicon bottle.

Among the objects in Fig. 5, the bevel gears and the pneumatic connector had complex outer surfaces which caused difficulty in forming contact with the wire. In Fig. 5a, the top bevel gear had two contact regions with the wire at the crown, and one contact region with the bottom bevel gear at the end-face, while the bottom bevel gear additionally had an extra contact with the wire at the flank surface. In Fig. 5b, originated from the non-uniform shapes, the wire only touched the pneumatic connector and the bottom bevel gear without contacting the top bevel gear at the initial state. Also, the contact between the pneumatic connector and the top bevel gear generated an unstable state for grasping in which the pose of the pneumatic connector was then rearranged under the compression of the wire. At the balance state, there was no correlative motion between the pneumatic connector and the top bevel gear during grasping, while the bottom bevel gear was still the most stable. The glue bottles in Fig. 5c had a longer body length and a smaller cross-section with a tapered shape. When the robot fingers approached and exerted the squeeze on these objects, the glue bottles began sliding on each other due to unstable contact between themselves. Due to the soft squeeze on the wire and the fingertips, such contact quickly reached a balanced state during manipulation. The double silicon bottles (see Fig. 5d) were the heaviest and the bulkiest among the object samples in this study. When the wire generated enough squeeze, these silicon bottles were stably balanced in the middle of the two fingers (or middle of FG and F′G′). The soft-elastic wire facilitated the silicon bottles to self-position prior to reaching the balance state.

**Figure 7:**
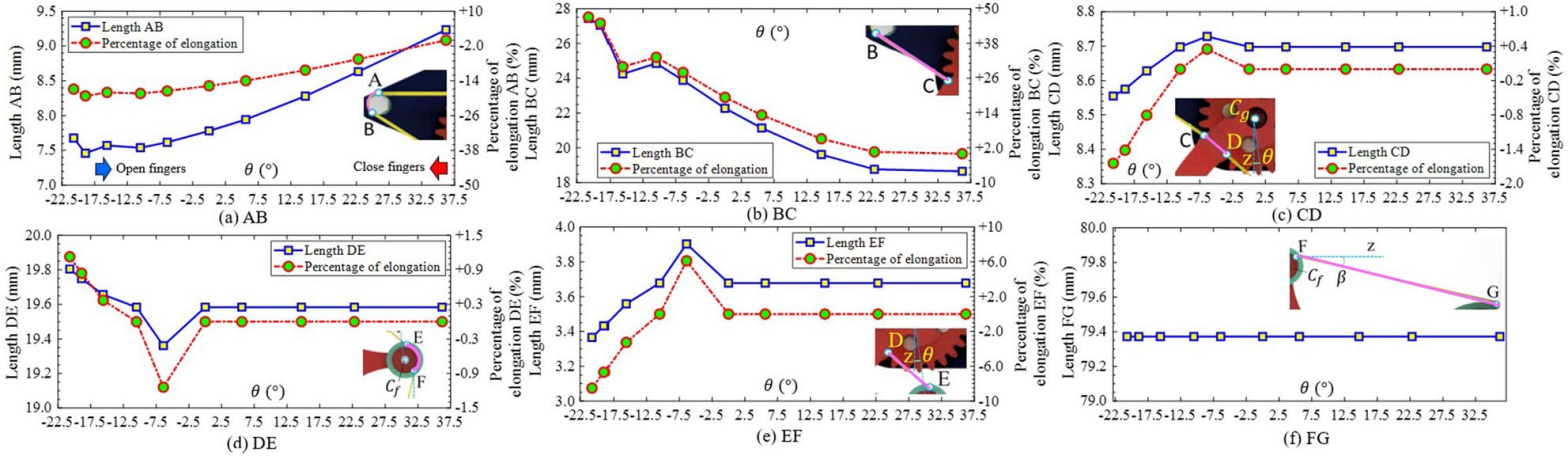
Changing the wire lengths at each line of the LH finger (also RH finger) with $\theta$ is a variation. In this figure, six investigated lines/curves include (**a**) AB, (**b**) BC, (**c**) CD, (**d**) DE, (**e**) EF, and (**f**) FG. Blue, and red big arrows in (**a**) indicated moving trends of the fingers: open, and close, respectively. Concurrently, the pink line in each inset image showed the investigating position of the wire.

The stability of manipulating multiple objects was also evaluated by iterating the test in Fig. 5 at different levels of $v_z$, denoted as a durability test for which the outcomes are shown in Fig. 6. On each graph, the successful cases presented the number of times the objects didn’t slip away from the fingers. At $v_z$ = 0.57, and 1.08 cm/s, our wiring-based gripper reached 100$\%$ successful cases after 10 iterations of the manipulation. When $v_z$ increased in the range of 0.57 to 3.63, the successful cases remained at 100$\%$ except for grasping the double glue bottles where the rate dropped down 60$\%$. Hence, the variation of the velocity in lifting partly affected the stability of the multiple objects, especially in the case of the double glue bottles. The failure trials were the consequence of the inertia of the grasped objects predominated in the total force on the manipulating process (Eq. 2).

**Figure 8:**
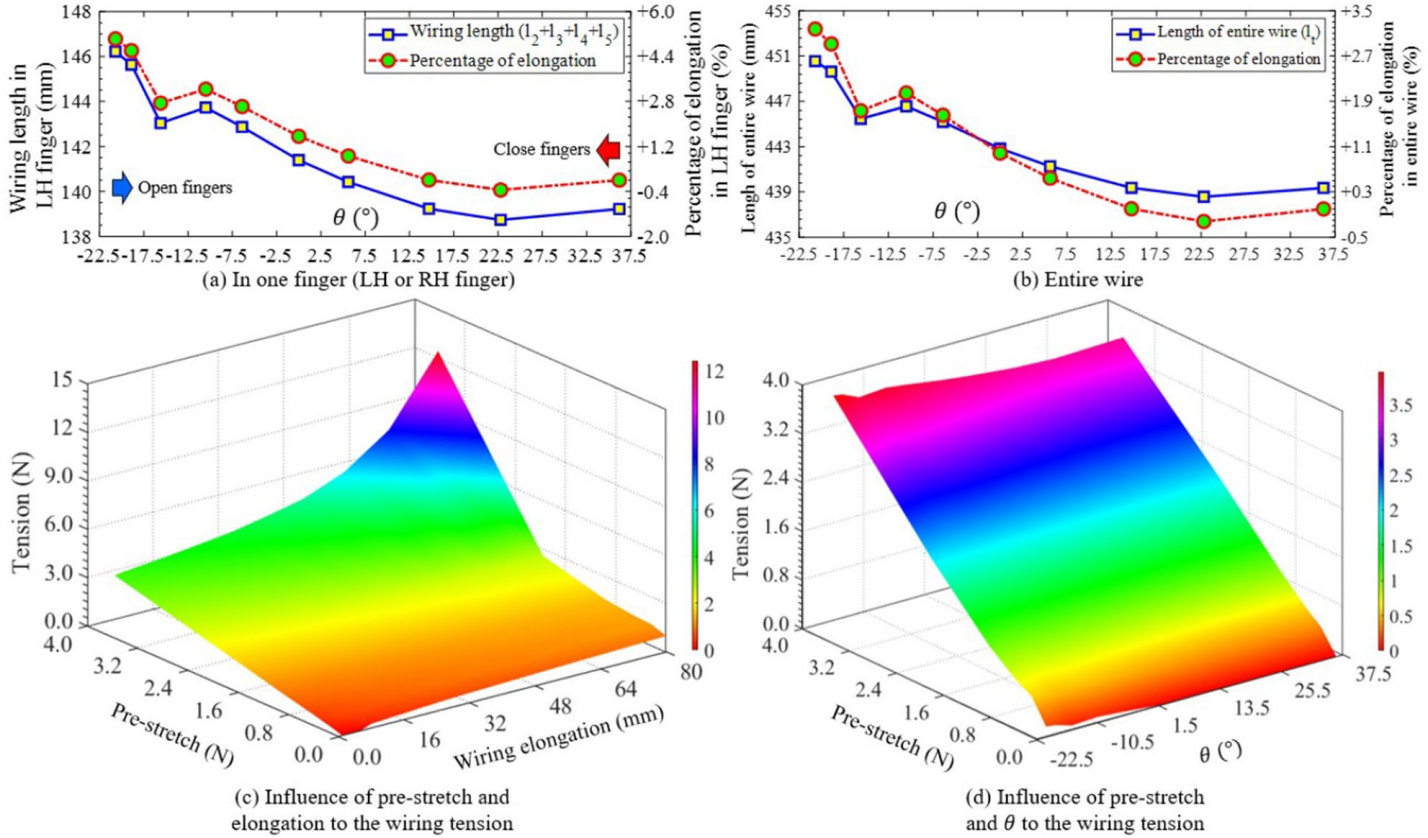
Wiring elongation in LH finger (RH finger) (**a**) and in the entire wire $l_t$ (**b**) with $\theta$ is a variation. Influence of the pre-stretch, and wiring elongation to the wiring tension (**c**) and pre-stretch and $\theta$ to the wiring tension (**d**).

The wiring elongation $\delta l$ directly influenced the wiring tension $^0_iT$ which exerted the squeeze on the objects. In our robot hand, the entire wire comprised multiple line/curve components as illustrated in Fig. 3. As two fingers opened or closed, the elongations in such components varied due to changing the contact positions between the wire and the finger skeleton. Figure 7 showed the wiring elongation at each line/curve component of the LH-finger according to the rotational angle $\theta$. The elongations of AB and BC respectively decreased from 7.68 to 7.46 mm (− 2.35$\%$), and from 27.44 to 27.05 mm (− 2.11$\%$) at $\theta = -20.7^{\circ }$, and $-\,18.9^{\circ }$ (see Fig. 7a,b). Then, these lines increased to 7.57 mm (1.2$\%$) and 24.25 mm (15$\%$) at $\theta = -15.6^{\circ }$ before AB and BC continuously went upwards to 9.23 mm (+ 18$\%$) and dropped 30$\%$ at the maximum $\theta$. At another pair, CD and EF initially became longer with the amounts of + 1.99$\%$, and +14.62$\%$ at $\theta = -20.7^{\circ }\rightarrow$ 36.2°, then fell down − 0.35%, and − 6.12% at $\theta = 0^{\circ }$ and remained unchanged until $\theta = 36.2^{\circ }$ (see Fig. 7c,e). DE originated at 19.81 mm continuously declined to 19.36 mm at $\theta = -6.4^{\circ }$ (see Fig. 7d). From here, this elongation went up to 19.58 mm (at $\theta = 0^{\circ }$) which was the constant value until the maximum $\theta$. There was no elongation for the line FG in the variation of $\theta$, where the length of FG kept an original value of 79.37 mm (see Fig. 7f). The maximum elongation of each line/curve [elongation mm ($100.^tl_n/^ul_n\%$)] AB, BC, CD, DE, EF, and FG were respectively 1.77 mm (19.19$\%$), 8.79 (47.1), 0.17 (2), 0.44 (2.27), 0.54 (14.63), 0 (0). Hence, BC, and FG had the biggest and smallest values of wiring elongations. Summing the elongations of 6 lines/curves in Fig. 7 deduced the elongation of the wire in the LH-finger and also in the RH-finger due to the symmetrical design. As shown in Fig. 8a, the wiring elongation in one finger was almost in a downward trend corresponding with the increment of $\theta$ except for two sudden rises at $\theta = -10.5^{\circ }$ and 36.2°. The maximum variation of the wiring elongation for this finger was 7.48 mm (5.37$\%$). Applying Eq. (6) into the entire wire, the total elongation had the same trends as the case of wire in one finger with the changing elongation increased double i.e. 14.96 mm (3.4$\%$) (see Fig. 8b). From these results, the more closing the fingers generated, the more rising tension the wiring tension the wire got.

The length of the entire wire reached a minimum value of 439 mm at $\theta = 22.8^{\circ }$, which was used as the base length for measuring the real tension (see Supplementary Fig. 1). Initially, one end of this wire was fixed at a mobile fulcrum that could slide in one direction, while the remaining end was attached to a force gauge fixed on the floor. When the mobile fulcrum moved, the wiring tension simultaneously changed values recorded on the force gauge. Measuring the tension was conducted at the pre-stretch of {0, 0.25, 0.97, 2, 3.63(N)} with the elongation in the range of 1$\rightarrow$80 mm. As shown in Fig. 8c, the wiring tension was in direct proportion with the pre-stretch and the wiring elongation. The tension values were bound to spike up at the large ranges of both the pre-stretch and the wiring elongation. At the same level of the pre-stretch, the variation of the wiring tension was {0.17, 0.2, 0.15, 0.1, 0.1, 0.38 (N)} respectively at the pre-stretch of {0, 0.25, 0.5, 0.97, 2, 3.63 (N)} (see Fig. 8c). Also, the tension slightly increased by the reduction of $\theta$ (or being towards the closing state of the fingers) (see Fig. 8d).

**Figure 9:**
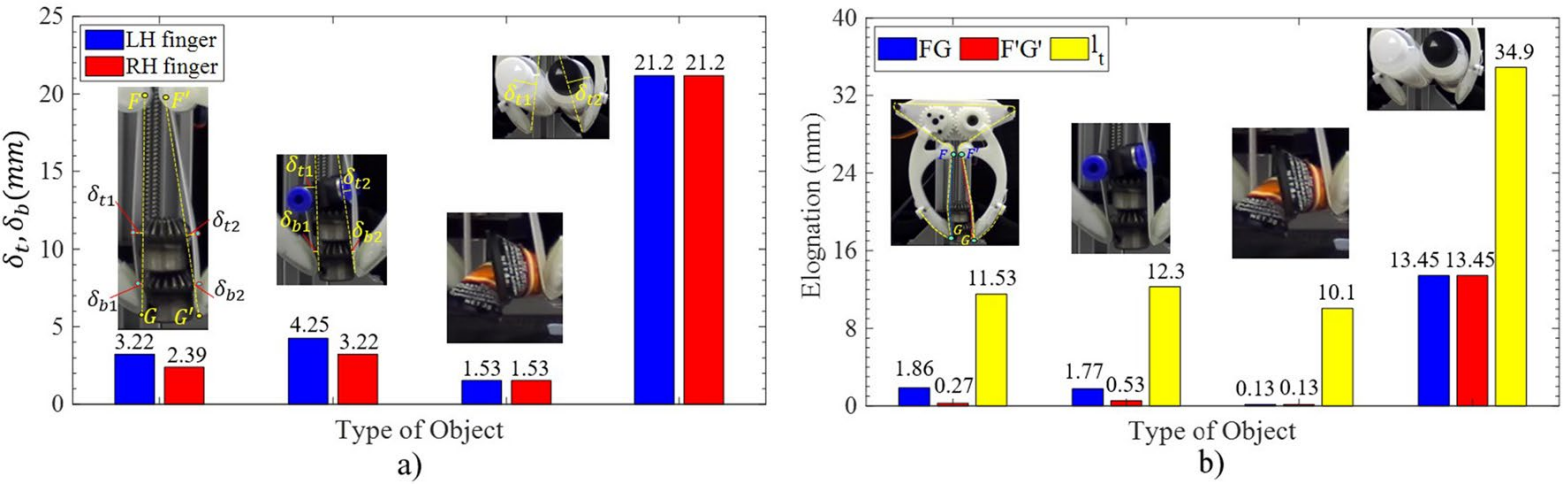
Comparisons of maximum wiring deflection of $\delta _{t1},\delta _{t2},\delta _{b1}$ and $\delta _{b2}$ (**a**) and the wiring elongation of FG, and F′G′ (**b**) between different types of the objects.

In Fig. 9a, $\delta$ ($\delta _{t1}, \delta _{t2},\delta _{b1}, \delta _{b2}$) placed the smallest and biggest values (1.53, and 21.2 mm) in the cases of grasping double glue bottles and double silicon bottles. Whereas, grasping the double bevel gears and double bevel gears plus a pneumatic connector deformed the wire with roughly 2 times and 2.4 times higher $\delta$ than trying the double glue bottles. The difference of $\delta$ between such cases originated from the object properties (shape, mass, surface properties), and the state contact (between wire-objects, fingertips-objects, and objects-objects). Also, $\delta$ was in direct proportion to the object mass and smaller than the limit deflection of 24 mm when the middle point of FG, and F′G′ touched finger skeleton. At the same trial, there was a slight difference of $\delta$ between RH- and LH-fingers in manipulating the double bevel gears and double bevel gears plus a pneumatic connector (roughly 1 mm). This led to the elongation differences of FG, and F′G′ corresponding with 1.6, and 1.24 mm (see Fig. 9b). Technically, the influence of the less than 2 mm elongation to the wiring tension was infinitesimal after collating the data in Fig. 8c. The tension significantly increased during grasping the double silicon bottles, since the total elongation of the wire was 34.8 mm (7.9$\%$).

## Conclusion

The experimental results clearly demonstrate that the wiring-based robot hand excels in picking up multiple specific objects in each trial, achieving highly successful rates. Across various levels of lifting velocity, the gripper demonstrated stable grasping with no failed cases, except for a few instances of poor results when grasping double glue bottles at the maximum velocity. Our gripper design has systematically addressed the key challenges faced by soft robot hands, namely the inefficiency or impossibility of grasping multiple objects in a single trial and instability when handling heavy loads. When compared to traditional grippers, the wiring-based robot hand stands out for its ability to generate a higher degree of flexibility and softer interactions, making it a promising advancement in the field of robotic manipulation.

### Supplementary Information

Supplementary Video 1.

Supplementary Information.

Supplementary Figure 1.
