# Author Correction: Soft-stable interface in grasping multiple objects by wiring-tension

**DOI:** 10.1038/s41598-024-53415-3

**Published:** 2024-02-05

**Authors:** Pho Van Nguyen, Dhyan Bohra Sunil, Wai Tuck Chow

**Affiliations:** 1 School of Mechanical and Aerospace Engineering, Nanyang Technological University, Singapore, Singapore; 2 Schaeffler Hub for Advanced Research Center (SHARE@NTU), Singapore, Singapore

Correction to: *Scientific Reports* 10.1038/s41598-023-47545-3, published online 06 December 2023

The original version of this Article contained an error in the name of author Wai Tuck Chow, which was incorrectly given as Tuck Wai Chow.

The original Article has been corrected.

