# Supplementary figures and images for: Soft-stable interface in grasping multiple objects by wiring-tension
Source: Sci Rep. 2023 Dec 6;13:21537. doi: 10.1038/s41598-023-47545-3 (PMC10700512; doi:10.1038/s41598-023-47545-3)

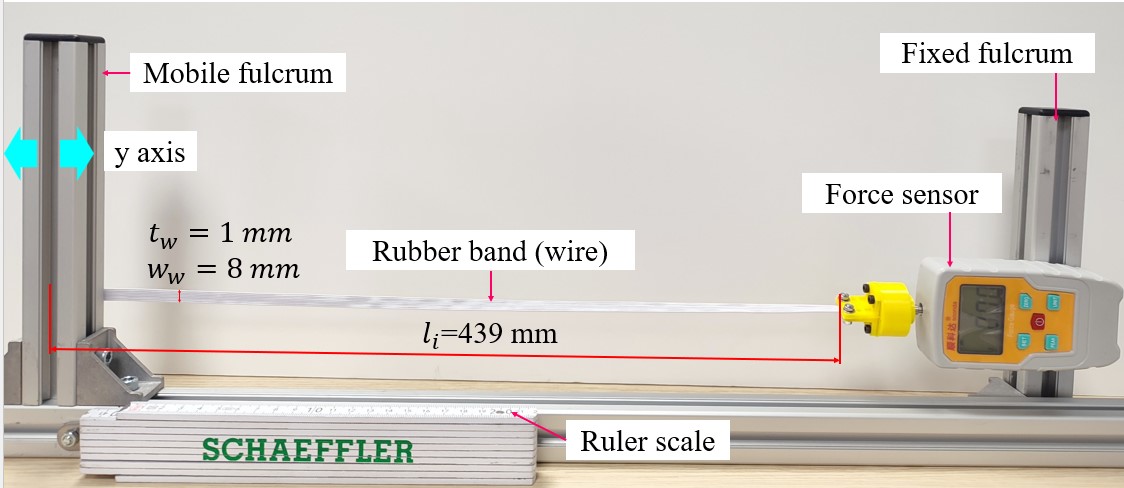

Supplement: Supplementary file 3 — Supplementary Figure 1. [file 41598_2023_47545_MOESM3_ESM.jpg]
